# Case report: Rapid recovery after intrathecal rituximab administration in refractory anti-NMDA receptor encephalitis: report of two cases

**DOI:** 10.3389/fimmu.2024.1369587

**Published:** 2024-03-06

**Authors:** Mahasen Reda, Rosette Jabbour, Asad Haydar, Fatima Jaafar, Nabil El Ayoubi, Omar Nawfal, Ahmad Beydoun

**Affiliations:** ^1^ Department of Neurology, American University of Beirut Medical Center, Beirut, Lebanon; ^2^ Division of Neurology, Saint George Hospital University Medical Center, Beirut, Lebanon

**Keywords:** case report, anti-N-methyl-D-aspartate receptor, encephalitis, refractory, intrathecal rituximab

## Abstract

**Background:**

Anti-N-methyl-D-aspartate receptor (anti-NMDAR) encephalitis is one of the most prevalent etiologies of autoimmune encephalitis. Approximately 25% of anti-NMDAR encephalitis cases prove refractory to both first- and second-line treatments, posing a therapeutic dilemma due to the scarcity of evidence-based data for informed decision-making. Intravenous rituximab is commonly administered as a second-line agent; however, the efficacy of its intrathecal administration has rarely been reported.

**Case summary:**

We report two cases of severe anti-NMDAR encephalitis refractory to conventional therapies. These patients presented with acute-onset psychosis progressing to a fulminant picture of encephalitis manifesting with seizures, dyskinesia, and dysautonomia refractory to early initiation of first- and second-line therapeutic agents. Both patients received 25 mg of rituximab administered intrathecally, repeated weekly for a total of four doses, with no reported adverse effects. Improvement began 2–3 days after the first intrathecal administration, leading to a dramatic recovery in clinical status and functional performance. At the last follow-up of 6 months, both patients remain in remission without the need for maintenance immunosuppression.

**Conclusion:**

Our cases provide evidence supporting the intrathecal administration of rituximab as a therapeutic option for patients with refractory anti-NMDAR encephalitis. Considering the limited penetration of intravenous rituximab into the central nervous system, a plausible argument can be made favoring intrathecal administration as the preferred route or the simultaneous administration of intravenous and intrathecal rituximab. This proposition warrants thorough investigation in subsequent clinical trials.

## 1 Introduction

Anti-N-methyl-D-aspartate receptor (anti-NMDAR) encephalitis, first described in 2007 (1), is one of the most prevalent etiologies of autoimmune encephalitis, with an annual incidence of 1.5 per million population (2). Predominantly afflicting young women, this condition is usually characterized by early-stage psychiatric and/or behavioral disturbances (2). As the condition evolves, patients may exhibit various neurological symptoms, including seizures, dyskinesias, cognitive dysfunction, and autonomic dysfunction (2).

A distinctive feature of anti-NMDAR encephalitis lies in its association with ovarian teratomas in a subset of cases. Approximately 37% of patients with this condition are reported to harbor ovarian teratomas, playing a role in the pathogenesis by hosting dysmorphic neurons expressing NR1/NR2A/NR2B antigens (3). This elicits the activation of germinal centers, leading to the production of NMDAR antibodies, resulting in the internalization and reversible reduction in the number of NMDAR, thus giving rise to the clinical manifestations of anti-NMDAR encephalitis (3).

Approximately 25% of anti-NMDAR encephalitis cases prove refractory to both first- and second-line treatments (4), posing a therapeutic dilemma due to the scarcity of evidence-based data for informed decision-making. In this context, we present two cases of severe and refractory anti-NMDAR encephalitis, characterized by an inadequate response to first- and second-line agents. These cases exhibited a rapid and striking clinical improvement after the intrathecal administration of rituximab, offering a novel and promising alternative for cases resistant to conventional treatments.

## 2 Case description

### 2.1 Case 1

A previously healthy 35-year-old woman presented to the emergency department with acute-onset behavioral changes following an upper respiratory tract infection. That same day, she woke up with new-onset delusions about imminent death, accompanied by disorientation, labile mood swings, and inappropriate laughter and crying. A brain MRI revealed a non-enhancing high T2 FLAIR signal in the left hippocampus. Cerebrospinal fluid (CSF) analysis showed lymphocytic pleocytosis (95 WBCs, 95% lymphocytic) with normal protein and glucose levels. Tentatively diagnosed with viral encephalitis, intravenous acyclovir was initiated, despite negative cultures and a negative herpes simplex virus (HSV) PCR.

Over the subsequent 2 days, her condition deteriorated, progressing to hallucinations, mutism, diffuse rigidity, and catatonia in addition to new-onset seizures. Based on the suspicion of autoimmune encephalitis, a 5-day course of 1-g methylprednisolone intravenously daily was administered while awaiting CSF and serum autoimmune antibody titers. However, her condition worsened and progressed to unresponsiveness with prominent orofacial dyskinesias and recurrent bouts of sympathetic hyperactivity in addition to frequent electrographic seizures identified on EEG. Intravenous immunoglobulin (IVIg) at a dose of 2 g/kg over 5 days failed to improve her clinical condition, necessitating intubation due to central hypoventilation syndrome. At that time, NMDAR antibody titers in the CSF returned positive with a titer of 1:32 on the day of admission and 1:128 on day 3 (Bioscientia Laboratories). Repeat brain MRI showed progression of the high FLAIR signal, involving the left parahippocampal gyrus. A pelvic MRI revealed a complex left adnexal mass, subsequently resected with pathology diagnostic of a mature cystic teratoma. Owing to the lack of clinical improvement, the patient underwent five sessions of plasma exchange therapy. Subsequently, she received two doses of 500 mg of intravenous rituximab, with each dose administered 1 week apart. Despite these interventions, there was evidence of clinical deterioration over the next 2 weeks, as she remained comatose with hypoventilation, worsening orofacial dyskinesias, and bouts of severe sympathetic hyperactivity, and required a tracheostomy.

At that point (day 38 of admission), the patient received 25 mg of rituximab intrathecally, repeated weekly for a total of four doses with no reported adverse effects. A remarkable clinical improvement ensued, with spontaneous eye opening on the second day after administration of the first dose, obeying simple commands on day 3, and complete resolution of orofacial dyskinesias. She attempted to verbalize on day 5 and showed daily improvement. The NMDAR antibody titer in the CSF drawn at the time of her third intrathecal rituximab administration was 1:16. The patient was discharged to a rehabilitation center on day 58 of her admission. At her last follow-up visit, approximately 7 months from symptom onset, the patient had a normal sensorimotor examination with a modified Rankin scale of 1. However, mild cognitive deficits in memory, attention, ability to calculate, and executive function were noted.

### 2.2 Case 2

A previously healthy 16-year-old woman presented to the emergency department with generalized convulsive status epilepticus accompanied by high-grade fever. Ten days before presentation, she began experiencing mood swings, anxiety, and fear, along with delusions and hallucinations. Her symptoms subsequently progressed to include memory difficulties and focal impaired awareness seizures initially misdiagnosed as functional seizures by an outside physician. A brain MRI revealed mild increase FLAIR signal over both mesial temporal areas and CSF analysis showed lymphocytic pleocytosis (50 WBC cells; 80% lymphocytes), with normal protein and glucose levels. Despite benzodiazepines, loading doses of levetiracetam, and valproate, the status epilepticus persisted, eventually being controlled with propofol infusion. Treatment with intravenous acyclovir was initiated in addition to a 5-day course of 1-g methylprednisolone intravenously based on suspicion of anti-NMDAR encephalitis while awaiting antibody titers.

Following the pulse steroid therapy, the patient’s condition worsened with persistent hallucinations, prominent orofacial dyskinesias, autonomic dysfunction, and breakthrough seizures following attempts of propofol taper despite the introduction of various antiseizure medications including phenytoin, lacosamide, perampanel, and clonazepam. Following IVIg administration at a dose of 2 g/kg over 5 days, there was no improvement in her overall clinical condition with persistence of the super refractory status epilepticus that failed to respond to the ketogenic diet. A total body CT scan and ultrasound of the ovaries were negative. Three weeks after presentation, the patient received a single intravenous infusion of cyclophosphamide, totaling 1,600 mg, concomitant with the intravenous administration of 500 mg of rituximab. Subsequently, the patient received three additional doses of intravenous rituximab, each at a dosage of 500 mg, spaced 1 week apart. However, because of the development of high-grade fever and sepsis, the patient did not receive any further cyclophosphamide administration. The NMDAR antibody titer in the CSF drawn on the day of admission was 1:32. The patient received 25 mg of rituximab intrathecally, repeated weekly for a total of four doses with no reported adverse effects. Two days following the first intrathecal injection, the patient was successfully weaned off the anesthetic with no seizure recurrence. In addition, the orofacial dyskinesias and autonomic dysfunction subsided. She gradually regained full alertness and was discharged to a rehabilitation center for the management of her critical care neuromyopathy. At her last follow-up, 6 months from symptoms onset, the patient was found to have a mild distal, symmetric sensorimotor deficit secondary to critical care neuropathy and mild memory deficit. She remained seizure free on levetiracetam administered as monotherapy.

## 3 Discussion

Our findings substantiate the efficacy and safety of intrathecal rituximab administration in patients with refractory anti-NMDAR encephalitis who fail to respond to first- and second-line therapeutic agents. Our two patients, who were in a comatose state and failed to respond to pulse steroids, IVIg, intravenous rituximab (patients 1 and 2), and oophorectomy and plasmapheresis (patient 1), exhibited a rapid and remarkable improvement in their mental status following the initial intrathecal rituximab administration. The intervention also led to the resolution of the severe orofacial dyskinesias (patients 1 and 2), dysautonomia (patient 1), and super refractory status epilepticus (patient 2), within 48 h.

Our results align with a few previously documented cases (**Table 1**) where intrathecal rituximab administration to refractory anti-NMDAR encephalitis patients resulted in a swift and substantial recovery (5–7). This underscores the therapeutic potential of intrathecal rituximab in cases where its intravenous administration has failed or when systemic administration is hindered by factors such as severe infections.

**Table 1:** Characteristics of patients who received intrathecal rituximab for refractory anti-NMDA encephalitis.

	Casares et al. (5)	Krishnan et al. (6)	Krishnan et al. (6)	Santiago et al. (7)	Santiago et al.* (7)	Case 1 (current report)	Case 2 (current report)
**Gender, age (years)**	F, 20	F, 15	F, 16	F, 4	F, 5	F, 35	F, 16
First-line agents							
**IVMP + IVIG**	√	√	√	√	√	√	√
**PLEX**	–	√	√	–	√	√	–
Second-line agents							
**IV rituximab**	√	√	√	√	√	√	√
**IV cyclophosphamide**	–	√	√	√	√	–	√
Persistent symptoms after second line							
**Uncontrolled seizures**	–	√	√	–	–	–	√
**Dyskinesia**	√	–	√	–	–	√	√
**Dysautonomia**	–	–	–	–	–	√	√
**Encephalopathy**	√	√	√	–	–	√	√
**Need for mechanical ventilation**	–	–	–	–	–	√	√
**mRS**	–	–	–	3	5	–	–
**Dose of IT rituximab**	25 mg weekly for 4 doses	25 mg weekly for 4 doses	25 mg weekly for 4 doses	25–100 mg repeated in 2–4 weeks	25–100 mg repeated in 2–4 weeks	25 mg weekly for 4 doses	25 mg weekly for 4 doses
**Time to 1st clinical improvement after 1st dose of IT rituximab (days)**	2	2	3	NA	NA	3	2
Improvement after IT rituximab							
**Full seizure control**	–	√	√	–	–	√	√
**Resolved dyskinesia**	√	–	√	–	–	√	√
**Resolved dysautonomia**	–	–	–	–	–	√	√
**Improvement of mental status**	√	√	√	–	–	√	√
**mRS**	–	–	–	1	5*	–	–
**Duration of follow-up (months)**	11	8	8	6	12	7	6
**Maintenance immunosuppression**	Mycophenolate	None	None	None	None	None	None
**Relapse**	None	None	None	**None**	**None**	None	None

F, female; IV, intravenous; IT, intrathecal; IVIg, intravenous immunoglobulin; IVMP, intravenous methylprednisolone; mRS, modified Rankin Score; NA, no available data; PLEX, plasma exchange therapy. * This patient was ultimately found to have a potential alternate etiology for her symptoms (compound heterozygous variants in VPS13D) (NM_015378.2) (p.S2199G) and (p.R2433H) (OMIM #607317).

The recommended initial therapeutic approach for anti-NMDAR encephalitis involves tumor removal when applicable, high-dose intravenous steroids, IVIg, and plasmapheresis. Approximately 53% of patients respond to first-line treatment, typically within 4 weeks of initiating therapy (8). For non-responders, second-line agents such as rituximab, targeting CD20-positive B cells, or cyclophosphamide, an immunosuppressive agent, improve symptoms in a proportion of patients by modulating the immune response (4). Nevertheless, up to 25% of patients remain refractory despite second-line therapies (4).

For patients unresponsive to second-line agents, no established guidelines exist, and recommendations are based on expert opinions, small series, or isolated case reports. Third-line agents are categorized into cytokine-based drugs (e.g., tocilizumab, interleukin-2, basiliximab, anakinra, and tofacitinib), plasma cell-depleting agents (e.g., bortezomib and daratumumab), and treatments targeting intrathecal antibody synthesis (e.g., intrathecal methotrexate, natalizumab, and intrathecal rituximab) (9).

In our patients, the decision to proceed with intrathecal rituximab administration was carefully made after thorough consideration of all alternative off-label treatments (9). A crucial consideration was the observation from previous published cases that the efficacy of intrathecal rituximab could be assessed within a matter of days, whereas the efficacy of other off-label treatments might not be evident for several weeks. Therefore, our rationale was to administer intrathecal rituximab initially and monitor for response within approximately a week after the first administration. If no response was observed within this time frame, we planned to transition to tocilizumab, as this agent appeared to hold the most promising efficacy among the off-label treatments (9).

In addition, several other key factors lead to the choice of intrathecal rituximab administration in our patients including the presumed pathophysiology of CNS manifestations in anti-NMDAR encephalitis, the pharmacokinetics of rituximab, and its safety following this route of administration. Plasma cells located within the perivascular, interstitial, and Virchow Robin spaces of the brain are believed to play a pivotal role in the intrathecal synthesis of antibodies associated with anti-NMDAR encephalitis (3). Additionally, the fact that only 1% of intravenously administered rituximab crosses the blood–brain barrier (BBB) stands as a critical factor influencing its efficacy as it will not be able to target B cells that have crossed the BBB to become antibody-secreting plasma cells that perpetuate the disease process (3, 10). The safety profile of intrathecal rituximab administration, a crucial consideration, was previously established across various clinical conditions. In patients with CNS lymphoma, lymphomatous meningitis, and some cases of progressive multiple sclerosis, the safety of intrathecal rituximab was demonstrated when it was administered in weekly or biweekly doses of 25 mg (11–13). Adverse effects at this low dosage were of mild intensity and included nausea, vertigo, and paresthesia (5, 11–13).

The rapid clinical improvement seen in our patients following intrathecal rituximab administration is surprising since after systemic administration, it typically manifests a therapeutic effect over a span of weeks. It is purported that the mechanism of action of rituximab involves several pathways, including direct signaling, complement-dependent cellular cytotoxicity, and antibody-dependent cellular cytotoxicity (14). For instance, studies have demonstrated a direct local anti-lymphoma effect of rituximab when injected directly into the CSF in patients with central nervous system lymphoma (14), suggesting a direct effect on targeted lymphocytes, possibly mediated by complement augmentation (14). Moreover, systemic infusion of rituximab in autoimmune nephritis resulted in a profound depletion of total B cells within 48 h in most cases (15). In the context of autoimmune encephalitis, the pathogenic mechanisms of anti-NMDAR antibodies involve crosslinking, internalization, and reversible reduction in NMDA receptor density (16). *In vitro* studies have shown that this reduction is reversible, with levels returning to baseline within 4 days after antibody removal (3).

In our cases, it is therefore plausible that intrathecal rituximab administration induced a rapid depletion of CD20 lymphocytes, potentially amplified by inflammatory cytokines or complement due to a leaky BBB. This swift depletion of a small B-cell compartment may have led to an initial increase in NMDA receptor clusters and synaptic currents, thus correlating with the observed rapid clinical improvement. Subsequently, sustained changes occurred after completion of treatment, with restoration of the NMDA receptor’s density and reduction in autoantibody levels. Therefore, the prompt response observed following intrathecal rituximab administration may be attributed to complex interactions between rituximab, B-cell depletion, inflammatory mediators, and NMDA receptor dynamics. The exact mechanism will need to be elucidated with further research to delineate the precise mechanisms underlying the therapeutic effects of intrathecal rituximab in anti-NMDAR encephalitis.

However, given that the autoimmune mechanism in anti-NMDAR encephalitis is likely initiated and promoted outside the CNS, the first- and second-line therapeutic agents administered to our patients might have contributed to their clinical recovery. Nonetheless, it remains uncertain whether a sequential or concurrent use of therapies targeting both the central and the peripheral components of the autoimmune response would achieve a synergistic therapeutic effect, potentially enhancing treatment efficacy and leading to a more comprehensive disease control. Therefore, further research is warranted to delineate the specific contributions of each therapeutic approach.

It is important to note that both patients’ families were extensively informed on all available options for refractory cases, including intrathecal rituximab, which had only been employed in a limited number of cases of anti-NMDAR encephalitis patients. Detailed explanations regarding why intrathecal rituximab was preferred over alternative treatments were provided during discussions with both families, alongside a thorough examination of potential adverse effects. Written informed consent was obtained from the families of both patients following these discussions.

## 4 Conclusion

In conclusion, our cases provide evidence supporting the administration of intrathecal rituximab as a therapeutic option for patients with refractory anti-NMDAR encephalitis. This recommendation is based on its efficacy, swift onset of action, cost-effectiveness, and favorable safety profile. Given the limited penetration of intravenous rituximab into the central nervous system, a plausible argument can be made favoring intrathecal administration as the preferred route or the simultaneous administration of intravenous and intrathecal rituximab. This proposition warrants thorough investigation in subsequent clinical trials.

## Data availability statement

The original contributions presented in the study are included in the article/supplementary material. Further inquiries can be directed to the corresponding author.

## Ethics statement

Written informed consent was obtained from the individual(s), and minor(s)’ legal guardian/next of kin, for the publication of any potentially identifiable images or data included in this article.

## Author contributions

MR: Writing – review & editing, Writing – original draft. RJ: Writing – original draft, Writing – review & editing. AH: Writing – review & editing. FJ: Writing – review & editing. NE: Conceptualization, Writing – review & editing. ON: Writing – review & editing. AB: Conceptualization, Supervision, Visualization, Writing – review & editing.
